# Microbiological Profile of the Upper and Lower Respiratory Tract of Suckling and Weaned Dairy Calves with Acute Respiratory Disease

**DOI:** 10.3390/vetsci11100493

**Published:** 2024-10-11

**Authors:** Alice Fernandes Alfieri, Juliana Torres Tomazi Fritzen, Carolina Yuka Yasumitsu, Amauri Alcindo Alfieri

**Affiliations:** 1 Laboratory of Animal Virology, Department of Preventive Veterinary Medicine, Universidade Estadual de Londrina, Londrina 86057-970, PR, Braziljufritzen@uel.br (J.T.T.F.); carolinayyasumitsu@gmail.com (C.Y.Y.); 2 Multi-User Animal Health Laboratory, Molecular Biology Unit, Department of Preventive Veterinary Preventive, Universidade Estadual de Londrina, Londrina 86057-970, PR, Brazil; 3 National Institute of Science and Technology for Dairy Production Chain (INCT–LEITE), Universidade Estadual de Londrina, Londrina 86057-970, PR, Brazil

**Keywords:** dairy cattle herd, bovine respiratory disease, bovine respiratory viruses, bovine respiratory bacteria, nasopharyngeal swabs, lung tissues

## Abstract

**Simple Summary:**

Bovine respiratory disease (BRD) is a significant health issue affecting calves in dairy herds worldwide. BRD is caused by viruses and bacteria affecting the upper (URT) and lower respiratory tracts (LRT). The microbiological profile of BRD can vary widely, so it is important to understand the causes of outbreaks to treat and prevent them effectively. This study examined the microbiological profile of nine BRD outbreaks in dairy calves from Paraná, southern Brazil. An analysis was conducted on lung tissue (LRT *n* = 12) and deep nasopharyngeal swab (URT *n* = 24) samples. Three herds showed a viral etiology, mainly bovine viral diarrhea virus (BVDV). In three herds, the cause was bacterial (*Mycoplasma bovis*), while in one herd, a mixed infection of BVDV and *M. bovis* was found. In two outbreaks, no primary agents for BRD were identified; only bacteria were identified as secondary agents. Viral and single infections were more common in the LRT, causing pneumonia and deaths. Mixed bacterial infections predominated in the URT, involving more than one species of bacteria or bacteria and viruses. In the assessed region, it is recommended that calves receive sufficient levels of passive immunity to BVDV. In cases of BRD, antibiotics targeting *M. bovis* are recommended for treatment.

**Abstract:**

Bovine respiratory disease (BRD) is a significant global health issue in cattle farming, leading to substantial economic losses. This study analyzed the microbiological profiles of BRD outbreaks in nine dairy cattle herds in southern Brazil. We examined 36 biological samples, including 24 deep nasopharyngeal swabs (NS) and 12 lung tissue, from 29 suckling and 7 weaned heifer calves with acute BRD. PCR and RT-PCR techniques were used to partially amplify the genes of five viruses and four respiratory bacteria. A total of 8 different microorganisms, 4 viruses (bovine viral diarrhea virus, *n* = 5; bovine coronavirus, *n* = 3; bovine alphaherpesvirus 1, *n* = 3; and bovine parainfluenza virus 3, *n* = 2), and 4 bacteria (*Pasteurella multocida*, *n* = 16; *Mycoplasma bovis*, *n* = 8; *Histophilus somni*, *n* = 7; and *Mannheimia haemolytica*, *n* = 4) were identified in 29 (80.5%) samples. Seven samples (four lung tissue and three NS) were negative for all the microorganisms. Mixed infections were more common (62.1%) than single infections (37.9%). Bacterial nucleic acids were more commonly co-detected in NS than in lung tissue. Nucleic acids from a single pathogen were more frequently detected in lung tissues than in NS. *M. bovis* was the only bacterium detected in the lower respiratory tract. Understanding the microbiological profiles of the respiratory tracts of dairy calves with clinical signs of BRD is crucial for implementing effective biosecurity measures to prevent BRD in suckling and weaned dairy heifer calves.

## 1. Introduction

Bovine respiratory disease (BRD) is a significant global health issue in cattle farming. In dairy cattle herds, BRD can affect suckling and weaned calves and adult cows [1,2]. Depending on morbidity and mortality rates, BRD can lead to substantial economic losses in dairy farming [3,4].

The etiology of BRD is complex and may involve aspects related to management, immunity, and infectious diseases [5]. Upper respiratory tract (URT) infections can predispose cattle to lower respiratory tract (LRT) infections, triggering pneumonia, which results in an unfavorable clinical picture [6,7].

Several microorganisms can compromise the respiratory tract of cattle through single infections and, more frequently, through mixed infections. The viruses most commonly involved in infections of the URT and LRT of cattle are bovine alphaherpesvirus-1 (BoAHV-1), bovine viral diarrhea virus (BVDV), bovine parainfluenza virus-3 (BPIV-3), bovine respiratory syncytial virus (BRSV), and bovine coronavirus (BCoV). The most common bacteria that cause respiratory infections in cattle are *Pasteurella multocida*, *Mannheimia haemolytica*, *Histophilus somni*, and *Mycoplasma bovis* [5,8,9].

The initial component of innate immunity is the mucociliary apparatus. Mucus in the URT and pulmonary airways is produced by submucosal glands and goblet cells, and the mucus protects against microbial pathogens and other harmful agents such as inhaled particulate matter, aerosols, and vapors. This mucus, associated with cilia, helps keep the deeper parts of the lungs sterile. Moreover, most bacteria and viruses in the respiratory system of cattle produce pathogen-associated molecular patterns (PAMPs). They are recognized by the cells lining the respiratory tract and alveolar and intravascular macrophages, which help to control infections [10].

In Brazil, the etiology of BRD is often determined based on individual clinical cases or specific outbreaks in beef feedlots or dairy cattle herds. In rare cases, simultaneous infections caused by multiple microorganisms, including bacteria, viruses, or both, have been evaluated [8,11]. Moreover, the diagnosis of some fastidious microorganisms, such as *M. bovis* and *H. somni*, is often neglected because of the difficulties in conducting the diagnostic procedure [12].

This report describes the microbiological profiles of BRD outbreaks in nine Brazilian dairy cattle herds.

## 2. Materials and Methods

### 2.1. Herds and Samples

The survey was performed on nine dairy cattle herds in the municipality of Carambei (24°55′04″ S, 50°05′50″ W) in the central–eastern mesoregion of Paraná State, southern Brazil. The herds included in the analysis were high-yielding, with more than 200 lactating Holstein cows under intensive management and reports of acute respiratory diseases in suckling and weaned calves. The affected calves displayed clinical signs such as nasal discharge, cough, dyspnea, lethargy, increased respiratory rate, depression, and increased temperature (>39.5 °C). All cows in the nine herds evaluated received vaccines produced with prototype strains of BoAHV-1, BVDV-1 and -2, BRSV, BPIV-3, *P. multocida*, and *M. haemolytica* every six months.

BRD outbreaks have occurred from April to August, spanning autumn and winter in Brazil. During this period, the region has average minimum and maximum temperatures of 11 and 19 °C, respectively, and an average rainfall of 82 mm (www.climatempo.com.br). Biological samples from the URT and LRT were collected by veterinarians and sent to the laboratory for etiological diagnosis of BRD. Soon after the collection of biological samples (deep nasopharyngeal swabs), all calves with clinical signs of BRD were treated with broad-spectrum antibiotics and nonsteroidal anti-inflammatory drugs. In total, 36 respiratory samples were sent, including 24 deep nasopharyngeal swabs and 12 lung tissues from suckling (*n* = 29) and weaned (*n* = 7) dairy heifer calves. All lung tissues were collected during necropsies of suckling calves that died of BRD.

### 2.2. Nucleic Acid Extraction

Lung tissue samples were mechanically disrupted using a TissueLyser LT (Qiagen, Hilden, Germany) and homogenized with 1 mL of sterile physiological solution (0.9% NaCl). After centrifugation at 1,000 rpm for 5 min, 500 µL of the solution was pretreated with 0.2 mg/mL proteinase K (Ambion, Grand Island, NY, USA) and sodium dodecyl sulfate at a final concentration of 1% (*v*/*v*). Nucleic acids from the lung tissue samples were extracted using a combination of phenol/chloroform/isoamyl alcohol (25:24:1) and silica/guanidine isothiocyanate [13]. Nucleic acid extraction from nasopharyngeal swab samples was performed using the silica/guanidinium isothiocyanate method [13].

### 2.3. Molecular Diagnostics

Viral RNA was detected using several molecular assays. We performed partial amplification of 288 bp of the 5′-untranslated region (5′-UTR) of BVDV by reverse transcription-PCR (RT-PCR) [14], 371 bp of the BRSV G gene by RT nested-PCR (RT-nPCR) [15], 647 bp of the BPIV-3 hemagglutinin-neuraminidase (HN) gene by RT-PCR [16], and 251 bp of the BCoV N gene by RT- semi-nested PCR (RT-snPCR) [17]. PCR was used to amplify 354 bp of the BoAHV1 gC [18], 460 bp of the *P. multocida* ORF clone KMT1 [19], 385 bp of the *M. haemolytica* lktA-artJ intergenic region [9], and 408 bp of the *H. somni* 16S gene [20], and nPCR assays were performed to amplify 488 bp of the *M. bovis* 16S–23S intergenic region [21]. Respiratory samples that showed positive results in the molecular assays confirmed by nucleotide (nt) sequence analysis of the amplicons were used as positive. Nuclease-free water (Invitrogen, Carlsbad, CA, USA) was used as the negative control in all PCR assays. The amplified products were analyzed by electrophoresis on a 2% agarose gel in TBE buffer (pH 8.4; 89 mM Tris; 89 mM boric acid; 2 mM EDTA), stained with ethidium bromide (0.5 μg/mL), and observed under ultraviolet light. A list of the primers used to target the specific genes of infectious agents associated with BRD is provided as a supplementary table (Table S1).

## 3. Results

In this study, nucleic acids from eight potential pathogens associated with the etiology of BRD were amplified from the nine microorganisms assessed. Using molecular techniques, such as PCR, nPCR, and RT-PCR, nucleic acids were amplified from at least one microorganism in 29 (80.5%) of the 36 biological samples tested. Among these, 8 (66.7%) of the 12 samples from the LRT and 21 (87.5%) of the 24 samples from the URT tested positive. Only 7 (19.4%) samples, including three from the URT and four from the LRT, yielded negative results for all the microorganisms evaluated. The most commonly detected microorganism was *P. multocida*, identified in 16 (76.2%) of the 21 positive samples obtained from the URT. In contrast, BRSV nucleic acid was not detected in any of the 24 deep nasopharyngeal swabs or 12 lung tissue samples.

The percentage of viral infections was higher in the respiratory samples from the LRT than in the URT samples, whereas *M. bovis* was the only bacterium found in the lung tissues. In contrast, bacteria were more commonly identified in URT samples compared to those in LRT samples. All four bacteria included in the diagnostic battery of this study were present in the nasopharyngeal swab samples, with particular emphasis on *P. multocida* and *H. somni*. Table 1 presents the microorganisms identified in the 29 positive samples, distributed according to the type (LRT or URT) of the sample analyzed and considering singular and mixed infections.

In the URT samples, singular and mixed bacterial infections were predominant (58.3%). Only 3 (12.5%) deep nasopharyngeal swab samples tested negative for nine potential pathogens of the bovine respiratory tract. In the LRT samples, singular viral (BVDV, BPIV-3, and BCoV) infections (33.3%) and viral/bacterial (BVDV and *M. bovis*) mixed infections (16.7%) were commonly observed. Among the samples obtained from the LRT, singular infections were the most common. Viral infections (BVDV, BPIV-3, and BCoV) were detected in four samples, and bacterial infections (*M. bovis*) were detected in two samples. In addition, two lung fragment samples showed mixed infections caused by virus (BVDV) and bacteria (*M. bovis*). Nucleic acids from the microorganisms included in the analysis could not be amplified from four lung tissue fragments.

Of the samples evaluated, 11 showed the presence of more than one type of bacterium and 5 samples showed bacterial and viral infections, making a total of 16 samples in which more than one microorganism was detected. In contrast, singular infections were found in only 5 nasopharyngeal swab samples, of which 2 samples tested positive for viruses (BoAHV-1 and BVDV) and three for bacteria (*P. multocida*, *M. haemolytica*, and *H. somni*). Table 2 shows the distribution of the results obtained for the 36 respiratory tract samples of suckling and weaned calves with BRD, according to the type of infection (single or mixed) and origin (LRT or URT) of the sample.

The frequency of BVDV, BCoV, and BPIV-3 detection in the nine dairy herds was low, and each of these viruses was detected in samples from only one herd. BoAHV-1 was detected in two herds. The bacteria were more widely distributed than viruses in the herds, with *P. multocida* predominant in seven of the nine herds evaluated.

The analysis of this study concerns biological material from BRD outbreaks, not an epidemiological survey. As a result, the numbers of the analyzed and positive samples and the microorganisms identified in the URT and LRT do not satisfactorily support the statistical analysis.

## 4. Discussion

This cross-sectional and retrospective study demonstrated the diversity of the microbiological profiles of the URTs and LRTs of suckling and weaned calves during BRD outbreaks. The calves belonged to nine high-yielding dairy cattle herds in the central–eastern mesoregion of Paraná State, southern Brazil. The diversity of microorganisms found in respiratory samples obtained from the LRT (lung) was lower than that observed in samples obtained from the URT (deep nasopharyngeal swabs). Infections caused by a single microorganism prevailed in the LRT group, whereas mixed infections with simultaneous detection of more than one bacterium or bacteria and viruses were common in the URT group. The presence of resident microbiota in the URT is associated with a high occurrence of mixed infections in the upper airways [22].

Oliveira et al. [8] evaluated the microbiological profile of a BRD outbreak that occurred in a dairy heifer calf-rearing unit in the state of Paraná. Molecular techniques (PCR, nPCR, and RT-PCR) were used to assess the microorganisms present in bronchoalveolar lavage fluid (BALF) collected from 21 calves (15 symptomatic and 6 asymptomatic) aged 6–90 days. Mixed infections (bacteria × bacteria and bacteria × viruses) were more commonly (72.2%) observed than those caused by a single microorganism (27.7%). Viruses were most frequently detected in BALF samples from symptomatic (BRD) calves. The frequency of diagnosis of the causative agent differed from that observed in the current study. In the BRD outbreak reported by Oliveira, et al. [8], the most common viral infection was BRSV (38.1%). In contrast, BRSV was the only virus not identified in the nine outbreaks described in our study. *P. multocida* was the most common bacterial infection observed in both studies. Oliveira, et al. [8] could not detect the genomes of BoAHV1, BPIV-3, and *M. haemolytica* in any of the BALF samples; however, these were detected in our study. In addition, similar to that observed in the BRD outbreak described by Oliveira, et al. [8], two bacteria (*H. somni* and *M. bovis*) were detected in suckling calves at frequencies of 19 and 33.3%, respectively. Notably, in Brazil, laboratory diagnoses of these bacteria in BRD are neglected.

A recent seroepidemiological survey in dairy cattle herds in the same dairy mesoregion revealed that BoAHV-1, BVDV, BPIV-3, and BRSV infections were endemic. Neutralizing antibodies against these viruses were detected in 71.4, 56.3, 96.8, and 63.4%, respectively, of the adult unvaccinated cows (*n* = 497) examined in 39 dairy cattle herds [23]. In the BRD outbreaks reported in our study, viral infections occurred more frequently in suckling calves than in weaned calves. Bacteria were identified in both the suckling and weaned calves. Regarding the type of material evaluated, both viruses and bacteria were identified in deep nasopharyngeal swabs. Viruses and bacteria were found in biological samples obtained from suckling calves, indicating that this age group, even in the presence of passive immunity, is at a high risk of infection and development of BRD. Aspects related to the cleaning and disinfection of facilities and utensils, management, animal welfare, and biosecurity should be rigorous during periods of high susceptibility of suckling calves. The implementation of a vaccination program for BRD control and prevention will also reduce the risk of clinical infection in suckling calves [4,24].

Notably, BVDV was the only virus present in biological samples obtained from weaned calves. This animal category probably no longer possesses passive immunity [25]. BVDV has an immunosuppressive potential [26]. The presence of BVDV infections in recently weaned calves in association with low titers or absence of passive immunity and the immunosuppressive characteristics of the virus might predispose calves to the development of URT or LRT infections; furthermore, BVDV infection in association with other bacterial and viral infections may lead to the development of BRD [7].

*M. bovis* infection in dairy cattle herds can lead to various clinical conditions, including reproductive failure, BRD, mastitis, and other less frequent issues [27]. In Brazil, the diagnosis of mycoplasmosis in dairy cattle herds is often neglected because of a lack of knowledge and difficulty in testing. However, our research group has shown that this pathogen shows a high frequency of occurrence in high-yielding dairy cattle herds in the central–eastern mesoregion of Paraná State [12]. In this survey, *M. bovis* was found in deep nasopharyngeal swabs and lung tissue samples obtained from suckling and weaned calves in four of the nine sampled herds. Notably, *M. bovis* was the only bacterium detected in LRT samples. *M. bovis* infection is a significant concern for animal health; this is because of the challenges in diagnosing and treating this infection as well as the lack of effective vaccines for infection control and prevention. These results show that in high-milk-production regions, *M. bovis* infection should be included in BRD diagnoses in suckling and weaned calves.

*H. somni* is another bacterium associated with a series of clinical signs in cattle, mainly reproductive failure [28]. Similar to that observed for *M. bovis* infections, the etiological diagnosis of histophilosis in dairy cattle herds is rarely performed [8,12]. *H. somni* nucleic acids were amplified from deep nasopharyngeal swabs obtained from suckling and weaned calves of three of the nine herds evaluated. This result shows that the bacterium is also present in dairy cattle herds in the mesoregion, highlighting its importance in the differential diagnostic batteries.

BRD is a significant health issue in heifer calf breeding that can affect milk production in compromised herds [3,5]. Considering that this study was conducted in the Brazilian region with the highest milk productivity per cow using high-yielding and high-genetic merit herds, the implementation of control and preventive measures against BRD is essential in the case of suckling and weaned calves to maintain the health of dairy herds. Management, hygiene, biosecurity, and animal welfare measures, together with a robust immunoprophylactic program, should be adopted and routinely monitored. Etiological diagnoses of BRD outbreaks must be performed frequently to define the etiology and establish treatment practices. In particular, detecting the presence of two important bacteria (*H. somni* and *M. bovis*) in young and adult animals from dairy cattle herds is crucial in these regions, because no commercial vaccine is currently available for infections caused by these bacteria.

This cross-sectional study involved the identification of the microbiological profile, which included viral and bacterial infections, in nine BRD outbreaks in suckling and weaned dairy heifer calves from high-performance dairy herds. The results of this study introduces a perspective for the development of longitudinal surveys that include both symptomatic and asymptomatic calves from vaccinated and unvaccinated dairy herds. Constructing the microbiological profile of different animal categories in herds with distinct health management conditions will enable more effective validation of BRD control and prevention measures for several health conditions identified in high-performance dairy cattle herds.

## Figures and Tables

**Table 1:** Microorganisms (viruses and bacteria) identified by molecular assays (PCR, RT-PCR, and nested-PCR) in the 29 positive samples from the lower and upper respiratory tract of suckling and weaned calves with bovine respiratory disease from nine dairy cattle herds, distributed according to the type of sample analyzed and considering singular and mixed infections, in the central–eastern mesoregion of Paraná State, southern Brazil.

Microorganism ^1^	Positive Samples (%)	
	LRT (Lung, *n* = 8) ^2^	URT (Nasal Swab, *n* = 21) ^2^
BoAHV1	-	3 (14.3)
BVDV	3 (37.5)	2 (9.5)
BPIV3	2 (25.0)	-
BCoV	1 (12.5)	2 (9.5)
*P. multocida*	-	16 (76.2)
*M. haemolytica*	-	4 (19.0)
*H. somni*	-	7 (33.3)
*M. bovis*	4 (50.0)	4 (19.0)

^1^ BoAHV1 (bovine alphaherpesvirus 1); BVDV (bovine viral diarrhea virus); BPIV3 (bovine parainfluenza virus 3); BCoV (bovine coronavirus); *P. multocida* (*Pasteurella multocida*); *M. haemolytica* (*Mannheimia haemolytica*); *H. somni (Histophilus somni); M. bovis* (*Mycoplasma bovis*). ^2^ Lower respiratory tract (LRT) and upper respiratory tract (URT).

**Table 2:** Distribution of all results obtained in the 36 samples from the respiratory tract of suckling and weaned calves with bovine respiratory disease, according to the type of infection (single or mixed) and origin (LRT or URT) of the sample.

Infection Type	Microorganism ^1^	No. Sample
	LRT ^2^ (Lung Fragments)	
Single	BVDV	1
	BPIV-3	2
	BCoV	1
	*M. bovis*	2
Mixed	BVDV + *M. bovis*	2
Negative		4
Subtotal		12
	URT ^2^ (nasopharyngeal swab)	
Single	BoAHV1	1
	BVDV	1
	*P. multocida*	1
	*M. haemolytica*	1
	*H. somni*	1
Mixed	*P. multocida + M. haemolytica*	2
	*P. multocida + H. somni*	5
	*P. multocida + M. bovis*	3
	*P. multocida +* BoAHV1	2
	*P. multocida +* BCoV	2
	*H. somni + M. bovis*	1
	*P. multocida + M. haemolytica +* BVDV	1
Negative		3
Subtotal		24
Total		36

^1^ BoAHV1 (bovine alphaherpesvirus 1); BVDV (bovine viral diarrhea virus); BPIV3 (bovine parainfluenza virus 3); BCoV (bovine coronavirus); *P. multocida* (*Pasteurella multocida*); *M. haemolytica* (*Mannheimia haemolytica*); *H. somni (Histophilus somni); M. bovis* (*Mycoplasma bovis*). ^2^ Lower respiratory tract (LRT) and upper respiratory tract (URT).

## Data Availability

The data presented in this study are available in the article.

## Supplementary Materials

The following supporting information can be downloaded at: https://www.mdpi.com/article/10.3390/vetsci11100493/s1, Table S1. List of primers with the corresponding target genome region used to detect infectious agents associated with bovine respiratory disease.
