# Supplementary material for: Microbiological Profile of the Upper and Lower Respiratory Tract of Suckling and Weaned Dairy Calves with Acute Respiratory Disease
Source: Vet Sci. 2024 Oct 11;11(10):493. doi: 10.3390/vetsci11100493 (PMC11512243; doi:10.3390/vetsci11100493)
Supplement: Supplementary file 1 [file vetsci-11-00493-s001.zip › vetsci-3207438-supplementary.pdf]

Table S1. List of primers with the corresponding target genome region used to detect infectious agents associated with bovine respiratory disease.

Organism	Abbreviation	Molecular technical	Genomic target	Amplicon (bp)	Reference
Bovine viral diarrhea virus	BVDV	RT-PCR	5′ untranslated region (5′-UTR)	288	Vilcek *et al.* [1]
Bovine respiratory syncytial virus	BRSV	RT-n-PCR	G attachment glycoprotein gene	371	Vilcek *et al.* [2]
Bovine parainfluenza 3 virus	BPIV-3	RT-PCR	Hemagglutinin-neuraminidase gene	647	Zhu *et al.* [3]
Bovine coronavirus	BCoV	RT-sn-PCR	Nucleocapsid protein gene	251	Takiuchi *et al.* [4]
Bovine alphaherpesvirus 1	BoAHV-1	PCR	Glycoprotein C gene	354	Claus *et al.* [5]
Pasteurella multocida	*P. multocida*	PCR	Open reading frame of the clone KMT1	460	Townsend *et al.* [6]
Mannheimia haemolytica	*M. haemolytica*	PCR	lktA-artJ intergenic region	385	Angen *et al.* [7]
Histophilus somni	*H. somni*	PCR	16S ribosomal (r)DNA gene	400	Angen *et al.* [8]
Mycoplasma bovis	*M. bovis*	n-PCR	16S-23S rDNA intergenic region	488	Voltarelli *et al.* [9]

1. Vilcek, S.; Herring, A.J.; Herring, J.A.; Nettleton, P.F.; Lowings, J.P.; Paton, D.J. Pestiviruses isolated from pigs, cattle, and sheep can be allocated into at least three genogroups using polymerase chain reaction and restriction endonuclease analysis. *Arch Virol* **1994**, *136*, 309-323, doi:10.1007/BF01321060.
2. Vilcek, S.; Elvander, M.; Ballagi-Pordany, A.; Belak, S. Development of nested PCR assays for detection of bovine respiratory syncytial virus in clinical samples. *J Clin Microbiol* **1994**, *32*, 2225-2231, doi:10.1128/JCM.32.9.2225-2231.1994.
3. Zhu, Y.M.; Shi, H.F.; Gao, Y.R.; Xin, J.Q.; Liu, N.H.; Xiang, W.H.; Ren, X.G.; Feng, J.K.; Zhao, L.P.; Xue, F. Isolation and genetic characterization of bovine parainfluenza virus type 3 from cattle in China. *Vet Microbiol* **2011**, *149*, 446-451, doi:10.1016/j.vetmic.2010.11.011.
4. Takiuchi, E.; Stipp, D.T.; Alfieri, A.F.; Alfieri, A.A. Improved detection of bovine coronavirus N gene in faeces of calves infected naturally by a semi-nested PCR assay and internal control. *J Virol Methods* **2006**, *131*, 148-154, doi:10.1016/j.jviromet.2005.08.005.
5. Claus, M.P.; Alfieri, A.F.; Folgueras-Flatschart, A.V.; Wosiacki, S.R.; Medici, K.C.; Alfieri, A.A. Rapid detection and differentiation of bovine herpesvirus 1 and 5 glycoprotein C gene in clinical specimens by multiplex-PCR. *J Virol Methods* **2005**, *128*, 183-188, doi:10.1016/j.jviromet.2005.05.001.
6. Townsend, K.M.; Frost, A.J.; Lee, C.W.; Papadimitriou, J.M.; Dawkins, H.J. Development of PCR assays for species- and type-specific identification of Pasteurella multocida isolates. *J Clin Microbiol* **1998**, *36*, 1096-1100, doi:10.1128/JCM.36.4.1096-1100.1998.

7. Angen, O.; Thomsen, J.; Larsen, L.E.; Larsen, J.; Kokotovic, B.; Heegaard, P.M.; Enemark, J.M. Respiratory disease in calves: microbiological investigations on trans-tracheally aspirated bronchoalveolar fluid and acute phase protein response. *Vet Microbiol* **2009**, *137*, 165-171, doi:10.1016/j.vetmic.2008.12.024.
8. Angen, O.; Ahrens, P.; Tegtmeier, C. Development of a PCR test for identification of Haemophilus somnus in pure and mixed cultures. *Vet Microbiol* **1998**, *63*, 39-48, doi:10.1016/s0378-1135(98)00222-3.
9. Voltarelli, D.C.; de Alcantara, B.K.; Lunardi, M.; Alfieri, A.F.; de Arruda Leme, R.; Alfieri, A.A. A nested-PCR strategy for molecular diagnosis of mollicutes in uncultured biological samples from cows with vulvovaginitis. *Anim Reprod Sci* **2018**, *188*, 137-143, doi:10.1016/j.anireprosci.2017.11.018.
